# Genetic diversity of *Plasmodium vivax* population before elimination of malaria in Hainan Province, China

**DOI:** 10.1186/s12936-015-0545-2

**Published:** 2015-02-14

**Authors:** Yu-Chun Li, Guang-Ze Wang, Feng Meng, Wen Zeng, Chang-hua He, Xi-Min Hu, Shan-Qing Wang

**Affiliations:** Hainan Provincial Centre for Disease Control and Prevention, Haikou, 570203 China

**Keywords:** *Plasmodium vivax*, *pvmsp*-1, *pvcsp*, *pvmsp*-*3α*, *pvmsp*-3*β*, Hainan province, Elimination malaria, China

## Abstract

**Background:**

Hainan Province is one of the most severe endemic regions with high transmission of *Plasmodium falciparum* and *Plasmodium vivax* in China. However, the incidence of *P. falciparum* and *P. vivax* has dropped dramatically since 2007 and a national elimination malaria programme (NEMP) was launched after 2010. To better understand the genetic information on *P. vivax* population before elimination of malaria in Hainan Province, the extent of genetic diversity of *P. vivax* isolates in Hainan Province was investigated using four polymorphic genetic markers, including *P. vivax* merozoite surface proteins 1, 3α, and 3β (*pvmsp*-1, *pvmsp*-*3α*, and *pvmsp*-*3β*) and circumsporozoite protein (*pvcsp*).

**Methods:**

Isolates of *P. vivax* (n = 27) from Hainan Province were collected from 2009 to 2010 and *pvmsp*-1 and *pvcsp* were analysed by DNA sequencing, respectively. Using polymerase chain reaction-restriction fragment length polymorphism were analysed in *pvmsp*-*3α*, and *pvmsp*-*3β*.

**Results:**

The DNA sequencing analysis on *pvmsp1* revealed that there were three allele types: Salvador-1 (Sal-1), Belem and recombinant (R) types. Among them, Sal-1 type was a dominant strain with eight variant subtypes (88.9%), whereas R- (3.7%) and Belem-type strains (7.4%) had one variant subtypes, respectively. All the isolates carried *pvcsp* with VK210 type accounting for 85.2% (23/27 isolates) and VK247 type accounting for 14.8% (4/27). Only type A and type B alleles were successfully amplified in *pvmsp*-*3α* gene, and a high level of polymorphism was observed in *pvmsp*-*3α*. Considering *pvmsp*-3*β* gene, type A was the predominant type in 17 isolates (63%), whereas type B was dominant in only ten isolates (37%).

**Conclusion:**

The present data indicate that there was high degree of genetic diversity among *P. vivax* population in Hainan Province of China during the pre-elimination stage of malaria, with 26 unique haplotypes observed among 27 samples.

## Background

*Plasmodium vivax* is one of the most widespread species of human malarial parasites in Asia, Central and South America, the Middle East, and parts of Africa. The malarial infection causes substantial economic loss around the world, and billions of people are at risk of malarial infection [1,2]. In China, the situation has changed quickly and reported cases of malaria have declined dramatically [3,4]. In 2010, the Chinese Government decided to embark upon the National Malaria Elimination Programme (NMEP) with a goal of eliminating malaria by 2015 in the majority of regions, with the exception of the border region in Yunnan Province, and to completely eliminate malaria from PR China by 2020.

Hainan Province was one of the most severe endemic areas with high transmission of *Plasmodium falciparum* and *P. vivax*. There were no reports of autochthonous *P. falciparum* malarial cases before the implementation of NMEP; however, *P. vivax* was still transmitted [5,6]. Before achieving the goal of eliminating malarial infection in Hainan Island, it is essential to understand the diversity of autochthonous malaria and to predict the origin and the spread of parasite variants within and between populations. However, so far the population structure of *P. vivax* is less well understood and the population genetics of *P. vivax* has not been systematically studied in Hainan Province, before the start of elimination campaigns [7]. In this study, the population diversity of *P. vivax* isolates from Hainan Province was evaluated using four polymorphic markers.

## Methods

### Study area

**Figure 1:**
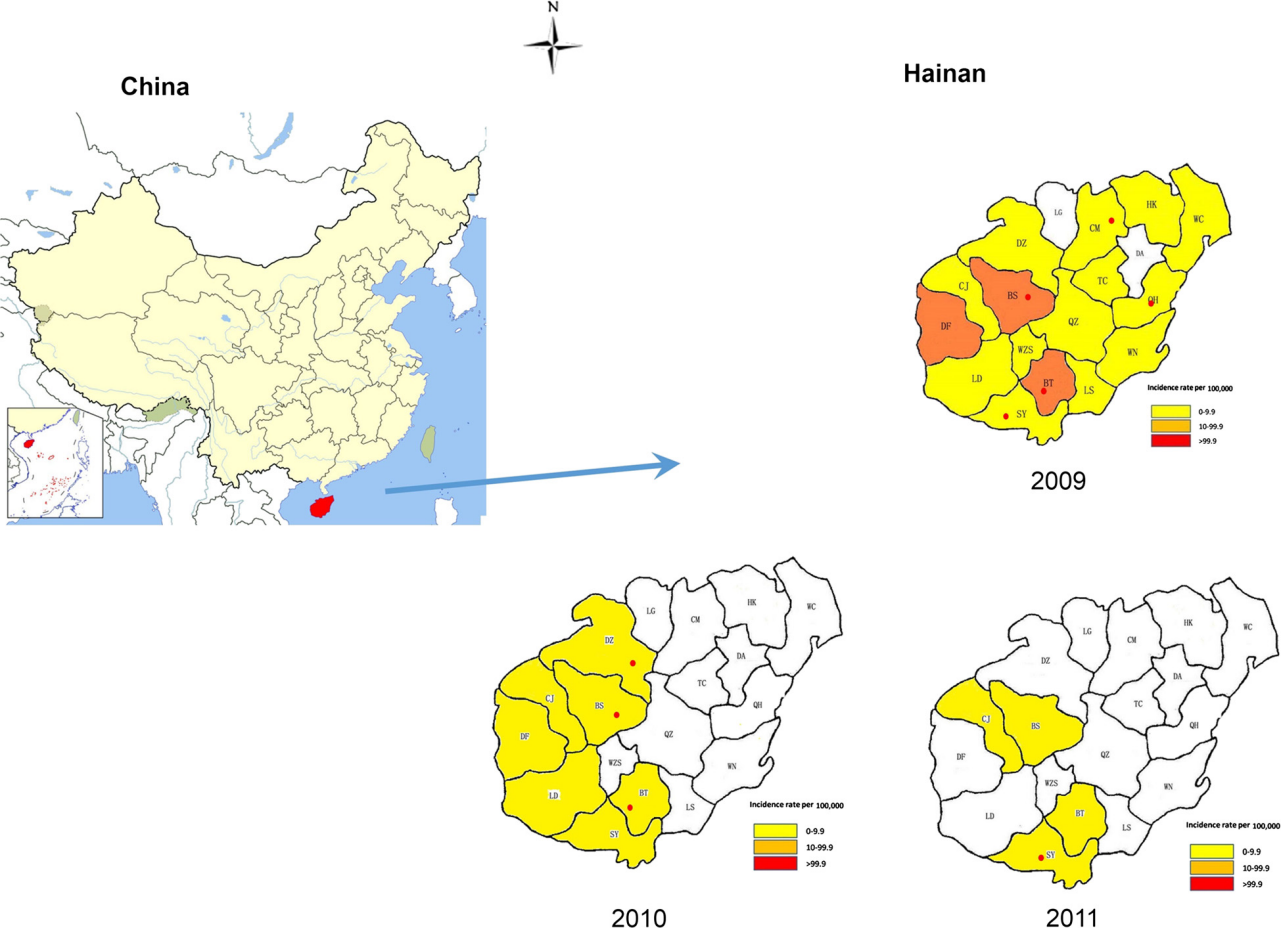
**The location of sample collection area in Hainan Province, PRChina, and the incidence and distribution of** ***Plasmodium vivax*** **between the year of 2009 and 2011.**

The study was conducted in the main island of the Hainan Province, which is located at north latitude 18°10′ - 20°10′ and east longitude 108°37′ - 111°03′, in southern China (Figure 1). The Province is characterized by mountainous and hilly landscape. The tropical monsoon and marine climate produce high temperatures and rich rainfall, which is suitable for malaria transmission [8]. Climatic and ecological conditions of this area make the environment favourable for mosquito breeding, and the main malaria vector are *Anopheles dirus* and *Anopheles minimus.*

### Patients and sample collection

Blood samples were collected from patients in Hainan Province who had symptoms of malaria from June 2009 to December 2012. The patients with malaria were diagnosed by blood smears and rapid diagnostic tests (RDTs) in township or county hospitals, before treatment [9]. All the samples were transported to Hainan Provincial Centre for Disease Control and Prevention (Hi CDC) for further confirmation by polymerase chain reaction (PCR) method. Of 226 reported malaria cases found by these methods, 37 cases were autochthonous *P. vivax*-infected patients, confirmed from malaria patient questionnaires. Two ml of whole blood sample was collected in EDTA and stored at −20°C until DNA extraction. This study was approved by the Ethics Committee of Hainan Provincial Center for Disease Control and Prevention (Hi CDC), China.

### DNA extraction

*Plasmodium vivax* genomic DNA was extracted from 80 μl of each infected blood sample using DNA blood kit following the manufacturer’s instructions (Qiagen, Germany) with minor modifications. The DNA was dissolved in TE buffer (10 mM Tris–HCl, pH 8.0, 0.1 M EDTA) and stored at −20°C until use. The quality of total DNA was analysed by running 5 μL of each DNA sample on a 1.0% agarose gel stained with ethidium bromide and visualized with UV illumination.

### PCR amplification of *pvmsp-3α*, *pvmsp-3β*, *pvmsp-1*, and *pvcsp* genes

To amplify the *pvmsp*-*3α*, *pvmsp*-3*β*, *pvmsp*-1, and *pvcsp* genes, a nested PCR amplification method was used following previously reported protocols with some minor modifications [10,11]. Oligonucleotide primers and cycling conditions are listed in Table 1. All amplification reactions were carried out in a total volume of 50 μL containing 20 μl of ddH_2_O, 2.0 μl of each primer (10 pM), 25 μl 2 × *pfu*Taq mixture, which contained 0.1 U *Pfu* polymerase/μl, 500 μM dNTP each, 50 mM Tris–HCl (pH 8.7), 20 mM KCl, 4 mM MgCl_2_, other stabilizing and strengthening reagents, following the manufacturer’s instructions (Tiangen, China). Primary amplification reactions were initiated with the addition of 2.0 μl of template genomic DNA prepared from the blood samples; 1.0 μl of the primary reaction amplification was used as template in the secondary amplification reactions. The amplified PCR products were resolved on 1.5% agarose gel, and the sizes of the PCR products were determined using a D2000 DNA ladder (Tiangen, China).

**Table 1:** **Primers for amplication on** ***pvmsp*** **-** ***3α*** **,** ***pvmsp*** **-3** ***β*** **,** ***pvmsp*** **-1, and** ***pvcsp*** **genes**

Gene Primers*	**Squencen(5′-3′)**	PCR cycling conditions**
*pvcsp*(N1)	F:5′-ATGTAGATCTGTCCAAGGCCATAAA-3′	95°C 3 min/[94°C 30 s, 58°C 30 s, 72°C 1.5 min] × 30 cycles, 72°C 5 min
	R:5′-TAATTGAATAATGCTAGGACTAACAATATG-3′	
*pvcsp*(N2)	F:5′-CCAGATGACGAGGAAGGAGATGC-3′	95°C 3 min/[94°C 30 s, 58°C 30 s, 72°C 1 min] × 35 cycles, 72°C 5 min
	R:5′-TCTTTCACAGACTTTTCATTTGGG-3′	
*pvmsp*-1(N1)	F:5′-GAGCCCTACTACTTGATGGTCC-3′	95°C 3 min/[94°C 30 s, 58°C 30 s, 72°C1min] × 35 cycles, 72°C 5 min
	R:5′-CCTTCTGGTACAGCTCAATG-3′	
*pvmsp*-*3α*(N1)	F:5′-CAGCAGACACCATTTAAGG-3′	95°C 3 min/[94°C 30 s, 54°C 30 s, 68°C 2.5 min] × 30 cycles, 68°C 5 min
	R:5′-CCGTTTGTTGATTAGTTGC-3′	
*pvmsp*-*3α*(N2)	F:5′-GACCAGTGTGATACCATTAACC-3′	95°C 3 min/[94°C 30 s, 55°C 30 s, 68°C 2.5 min] × 40 cycles, 68°C 5 min
	R:5′-ATACTGGTTCTTCGTCTTCAGG-3′	
*pvmsp*-3***β***(N1)	F:GTATTCTTCGCAACACTC	95°C 3 min/[94°C 30 s, 54°C 30 s, 68°C 2.5 min] × 30 cycles, 68°C 5 min
	R:CTTCTGATGTTATTTCCAG	
*pvmsp*-3***β***(N2)	F:CGAGGGGCGAAATTGTAAACC	95°C 3 min/[94°C 30 s, 55°C 30 s, 68°C 2.5 min] × 40 cycles, 68°C 5 min
	R:GCTGCTTCTTTTGCAAAGG	

N1 = Nest 1 (Primary) reaction; N2 = Nest 2 (Secondary) PCR reaction.

*F = Forward primer; R = Reverse primer. The reference sources of the primers are indicated.

**The cycling conditions have been modified in the present work.

The two columns indicate conditions for the primary and secondary amplification reaction.

### Sequence analysis and phylogeny on *pvmsp*-1, *pvcsp* genes

The nested PCR products of *pvmsp*-1, *pvcsp* were directly sequenced in both directions using an ABI PRISM3730 DNA sequencer by Sangon Biotech (Shanghai, China). Nucleotide or amino acid sequences obtained from the blood samples of the malaria patients in this study were compared by CLUSTAL X and BioEdit 5.0 programme with the following published sequences: VK210 (accession no. M28746) and VK247 (accession no. M28745) of *pvcsp* and Sal-I (accession no. M75674) and Belem (accession no. M60807) of *pvmsp*-1, respectively. Sequence relationship trees of the *pvmsp*-1 and *pvcsp* genes from the Hainan isolates and published sequences from isolates of different geographic locations of world were constructed using neighbour-joining method implemented in MEGA 3 program [12]. Bootstrap proportions were used to assess the robustness of the tree with 1,000 bootstrap replications.

### PCR/restriction fragment length polymorphism analysis of *pvmsp*-*3α* and *pvmsp*-3*β* gene

For restriction fragment length polymorphism (RFLP) analysis of *pvmsp*-*3α* gene, the PCR products were digested individually with restriction enzymes *Hha* I and *Alu* I in 20 μl reaction volumes at 37°C for three hours, as previously described [13]. Briefly, all digestion reactions were carried out in the presence of 10.0 μl PCR product, 2.0 μl of ddH2O, 1.0 μl of enzymes *Hha* I or *Alu* I (5 U/μl), and 2.0 μl of buffer according to the manufacturer’s instructions (TaKaRa, Japan). After electrophoresis on 2.5% agarose gel, the enzyme-digested fragments were visualized under UV illumination. The sizes of the digested fragments were estimated using 50 bp and D2000 ladder of molecular weight markers. The results were recorded and analysed on a Gel Doc XR image analyzer using Imagine Lab 2.0 software (Bio-Rad, USA). For RFLP analysis of *pvmsp*-3*β* gene, the reaction condition and ingredient of mixture except enzyme *Pst* I were the same as the RFLP analysis of *pvmsp*-*3α* gene.

## Results

### *pv*MSP-1 gene analysis and clustering

**Figure 2:**
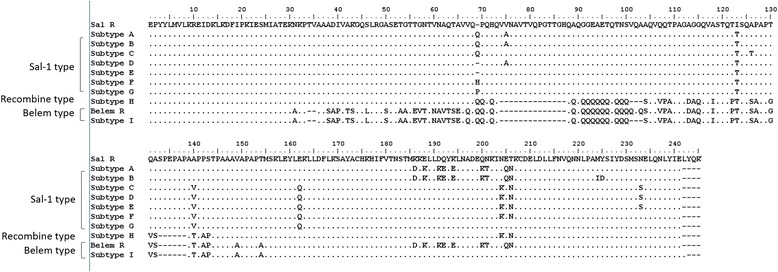
**Alignment of amino acid sequences of 9 pvmsp-1 distinct allelic variants and frequencies of pvmsp-1 allelic variants identified from 27 P. vivax Hainnan isolates.**

All 27 isolates from Hainan were successfully amplified for *pvmsp*-1 gene. Sequence analyses of the PCR products showed that the isolates could be divided into three types: Sal-I type, Belem type and recombination (R) type (Figure 2). Diverse non-synonymous substitutions were shown and distributed into nine different sequence types. Subtype A and subtype B showed the continually amino acid sequence substitution (DKKLLKEYE) specific to the Belem type. In other Sal-1 subtypes, seven amino acid substitutions (V/A, I/T, A/T, A/V, E/Q, N/K, T/N) were identified at different positions and only one glutamine or proline or histidine insertion. Comparing sequence with Belem reference, subtype I was classified as the Belem type and contained 14 poly-Q repeats and the amino acid sequence MKKELLDQYK specific to the Sal I type rather than the DKKLLKEYE specific to the Belem type. Comparison of the Sal-1 and Belem strains detected only one subtype (subtype H) of the recombinant strain, which displayed a forward and reverse sequence pattern similar to the Sal I and Belem strains, respectively. In addition to these sources of diversity, 13 glutamine residue repeats were present in this recombinant subtype.

In those types, Salvador-1 (Sal-1) was dominant strain and seven variant subtypes for the Sal-I type (88.9%). R-type (3.7%) or Belem-type (7.4%) only had one variant subtypes, respectively. In Sal-I subtypes, subtype A and subtype F were the most prevalent and accounted for 22.2%. The frequencies of others subtypes variants were evenly distributed among the variants.

**Figure 3:**
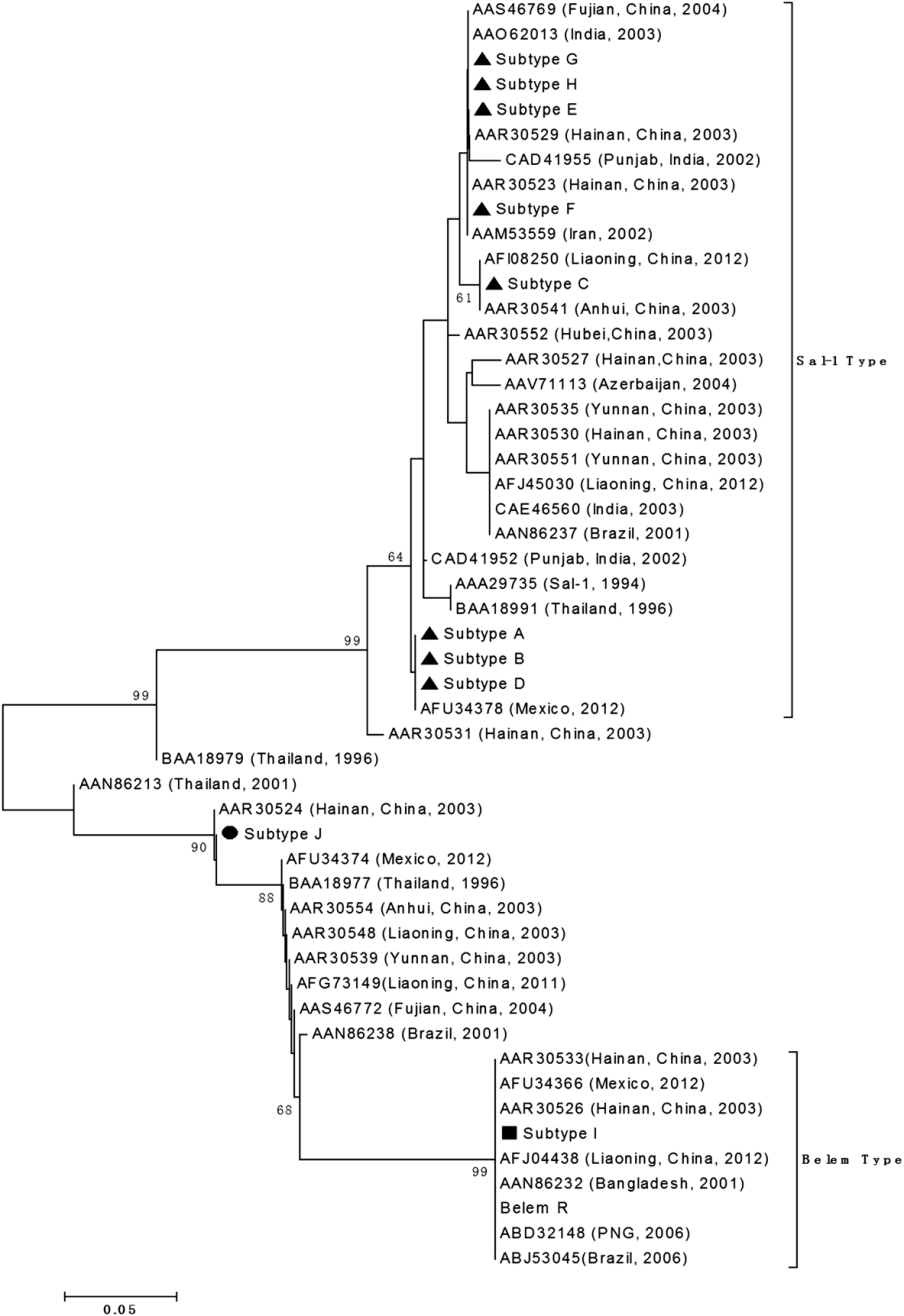
**Phylogenetic analysis of the P. vivax MSP-1 gene.** The phylogeny tree was constructed with the neighbor-joiningmethod using the MEGA 3 program.

A phylogenetic tree of the *pvmsp*-1 was constructed using amino acid sequences from the Hainan isolates and 54 published *pvmsp*-1 sequences obtained from the isolates of different geographic locations of world (19 from different provinces of China, one from Bangladesh, one from Azerbaijan, four from India, one from Iran, one from Papua New Guinea, three from Brazil, three from Mexico, and four from Thailand including the Sal- I strain and Belem reference strains). This analysis grouped the *P. vivax* Hainan population into two major clusters (Sal-I type and R-type) with different subtypes in each group (Figure 3). All of the *P. vivax* isolates examined in this study showed extremely high identity (range from 99 to 96%) with the isolates from different geographic locations of world. Subtype A and subtype B identified in this study were similar (99%) to India (Punjab) isolate (CAD41952), respectively. Subtype C and subtype F had extensive similarity (98%) with the Thailand isolate (BAA18985). Subtype D and subtype E were similar to the Thailand isolate (BAA18985), with 98 and 99% sequence homology, respectively. Similarity between subtype G and the Mexico isolate (AFU34378) was 98%. The recombinant strain (subtype H) had similarity (99%) with the Mexico isolate (AFU 34374) and Belem strain (subtype I) also had extreme similarity (96%) with the Mexico isolate (AFU34366).

### *pv*csp gene analysis and clustering

**Figure 4:**
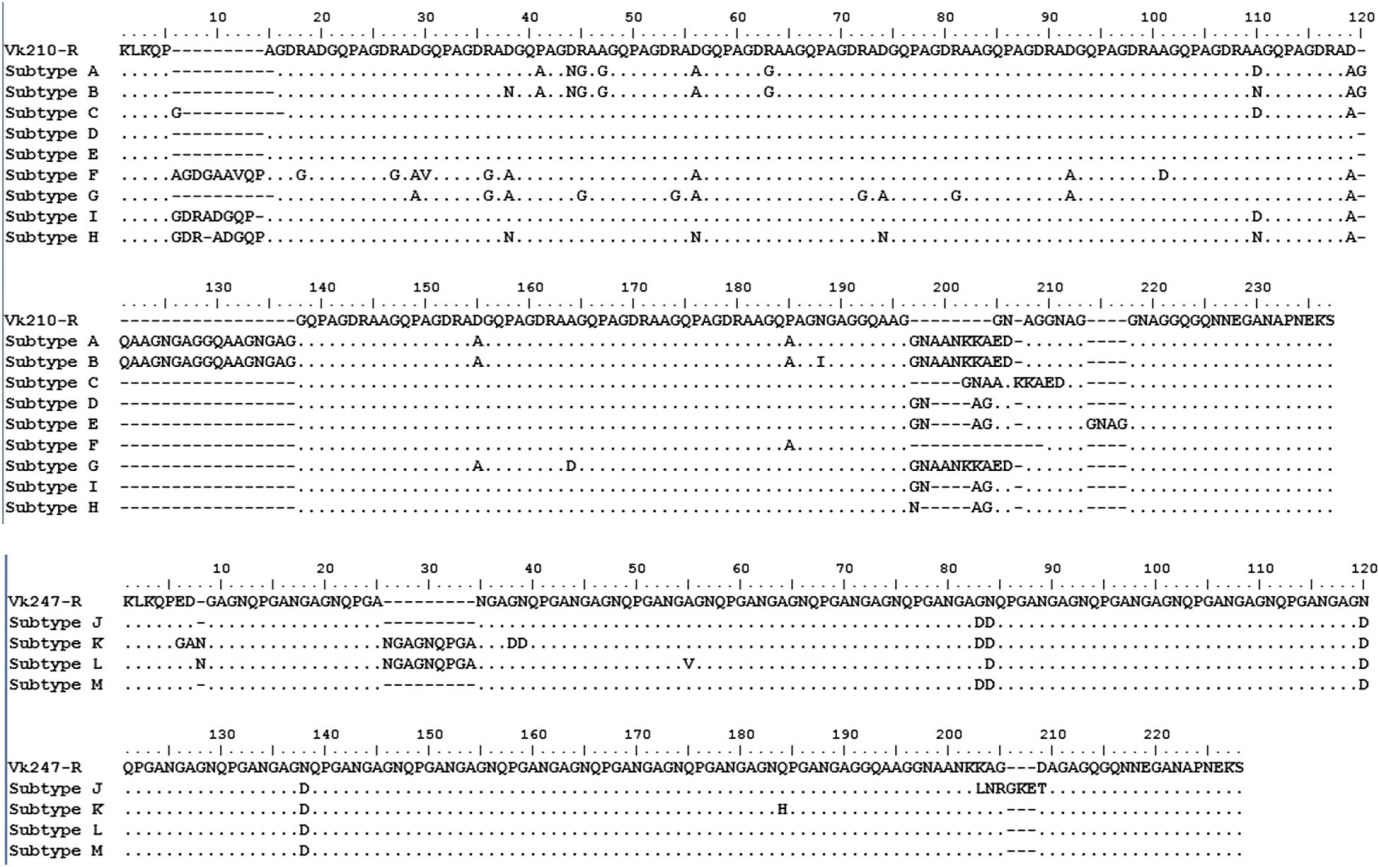
**Alignment of amino acid sequences of 13 Pvcsp distinct allelic variants and frequencies of Pvcsp allelic variants identified from 27 P. vivax Hainnan isolates.**

The classic *P. vivax* VK210 strain has a *pv*csp sequence that includes a GDRA(A/D)GQPA amino acid repeat. A variant form, VK247, later identified in Thailand, has an ANGAGNQPG amino acid repeat in the tandem repeat amino acid region [14]. Sequences of *csp* gene revealed that they were single infections in this study, not mixed infection. The majority of the isolates (85.2%, 23/27) were of the VK210 type while VK247 type accounted for 14.8% (4/27). Depending on the type and number of repeat motif, all of the DNA sequences belonged to VK210 type with nine distinct variants, and to VK247 type with four variants, whereas no *P. vivax*-like types were detected within these isolates. The frequency distribution of the 13 subtypes is shown in Figure 4a and b. Of the 13 subtypes, the most prevalent sequence variant was subtype F (25.9%, 7/27), followed by subtype A (18.5%, 5/27), subtype E and subtype G (11.1%, 3/27). The remaining nine subtypes of *pvcsp* gene were evenly distributed among these isolates.

**Figure 5:**
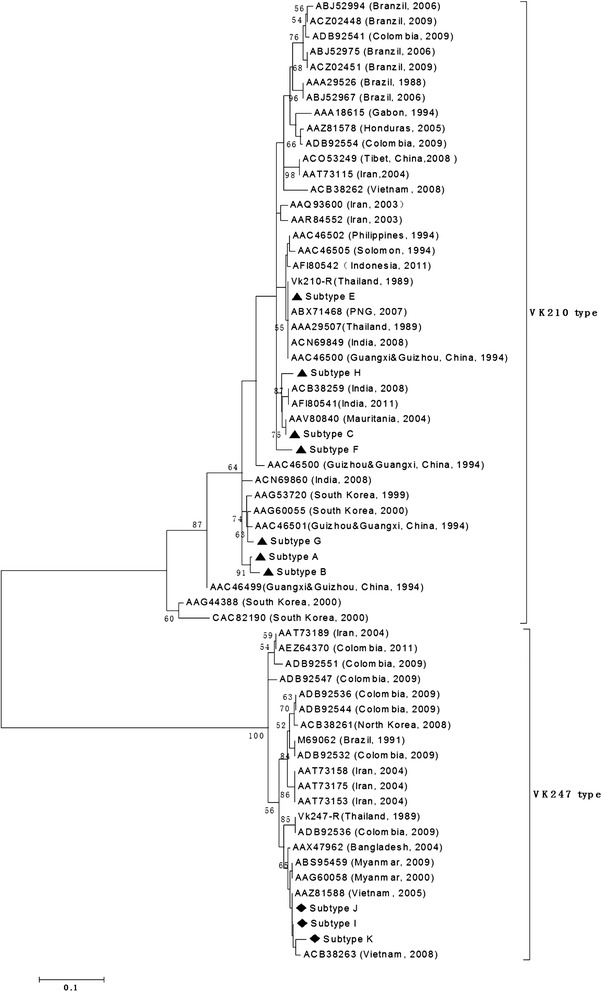
**Phylogenetic analysis of the P. vivax CSP gene.** The phylogeny tree was constructed with the neighbor-joiningmethod using the MEGA 3 program.

All variants started with the same pre-repeat sequence (KLKQP Region). The isolates displayed variations in the central peptide repeat motifs GDRA (A/D) GQPA or ANGAGNQPG with alternations of the repeating units. Each amino acid sequence group was representative of one or more strains. A phylogenetic tree of the *pvcsp* sequences was constructed using the neighbour-joining method based on the amino sequences from Hainan isolates in this study, and 51 published *pvcsp* sequences from the isolates of different geographic locations of world (five from China, one from Indonesia, three from India, seven from Iran, two from Myanmar, one from North Korea, four from south Korean, one from Papua New Guinea (PNG), one from the Solomon Islands, three from Vietnam, one from Honduras, one from Gabon, eight from Colombia, seven from Brazil, one from Philippines, two from Thailand, one from Bangladesh, one from Mauritania and two from Myanmar) (Figure 5). The sequences clustered into two distinct groups for the VK210 and VK247 type. And the analysis clearly showed that all subtypes of *pvcsp* from Hainan isolates clustered with the VK210 type and VK247 type in the tree, respectively. Some of the *P. vivax* isolates examined in this study showed 100% identity with strains from other regions of China or even from other countries, including China’s Tibet (AAA29534 with subtype A (VK210 type)), Mauritania (AFI80543 with subtype C (VK210 type)), Cambodia (AGN05237 with subtype D (VK210 type)). The remaining subtypes were new alleles identified in this study.

### PCR/RFLP analysis of the *pvmsp-3α* gene

**Figure 6:**
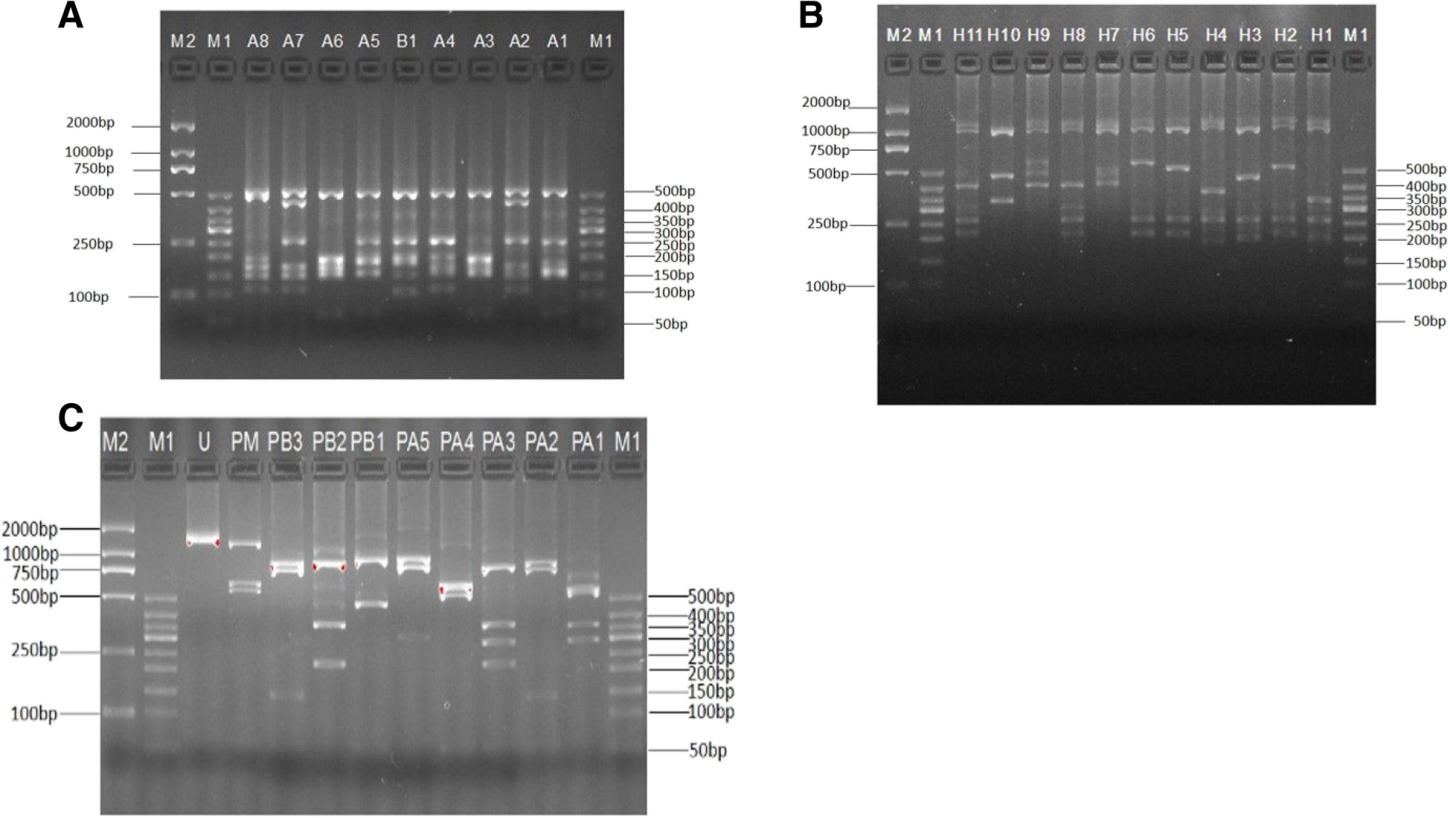
**PCR/RPLF genotypes and allelic frequencies of 27 P. vivax Hainan isolates based on Pvmsp-3α and Pvmsp-3β. (A)** The amplification products of Pvmsp-3α observed in Hainan P. vivax isolates were digested by *Alu I*, and nine variants were obtained (A1 to A8, B1). **(B)** The amplification products of Pvmsp-3α observed in Hainan P. vivax isolates were digested by *Hha I*, and eleven variants were obtained (H1 to H11). The lane with the molecular weight marker (50 bp ladder) is labeled as M1 and that of named D2000 marker do as M2. **(C)** The amplification products of Pvmsp-3β observed in Hainan P. vivax isolates were digested by *Pst I*, and ten variants were obtained (PA1 to PA6, PB1 to PB3, and U). The lane with the molecular weight marker (50 bp ladder) is labeled as M1 and that of named D2000 marker done as M2.

The *pvmsp*-*3α* gene was successfully amplified from all the 27 *P. vivax* isolates examined. Twenty-six samples of the PCR products from *P. vivax* isolates were the same size, approximately 1.9 kb (type A); one sample belonged to type B (~1.5 kb); no type C (~1.1 kb) or type D (~500 bp) was detected. The PCR-RFLP analysis revealed a high level of polymorphism in *pvmsp*-*3α* gene after the PCR products were digested with restriction enzymes *Hha* I and *Alu* I: nine (A1 to A8, B1) and 11 (H1 to H11) (Figures 6A, B). The frequency distribution of each variant is shown. In the *Alu* I digestion, allelic variants A1, A3 and A6 were the most common patterns, with frequencies of 29.6, 25.9 and 14.8%, respectively. Similarly, 70.4% of the *Hha*I digested samples were allele variants of H1, H2 and H3, representing 11.1, 29.6 and 29.6%, respectively.

### PCR/RFLP analysis of the *pvmsp*-*3β* gene

The *pvmsp*-*3β* gene was successfully amplified in 27 malaria samples and A type (1.7-2.4 kb) and B type (1.4–1.5 kb) were identified, following an earlier scheme [15]. Type A was the predominant type in 17 isolates (63%), while Type B accounted for only ten isolates (37%). Restriction analysis of the PCR products by *Pst* I revealed six variants (A1-A6) for type A allele and two variants (B1-B3) for type B allele, whereas mixed infection and undigested variants had one and eight, respectively (Figure 6C). In digestion, allelic variants PA1, PA2 and undigested were the most common patterns, with frequencies of 22.2, 18.5 and 29.6%, respectively. Other allelic variants were distributed evenly. Mixed infection with *pvmsp*-*3β* was considered in four isolates when more than one PCR product of different size was detected in a single sample, or when the summed size of the restriction fragments exceeded the size of the PCR products. The variant of PM (mixed infection) have three bands, the sizes of two bands exceeded 500 bp and that of the other band was near 2,000 bp. The summed size of the restriction fragments exceeded the size of the PCR products.

### Haplotypes from combined *pvmsp-1, pvcsp, pvmsp-3α and pvmsp-3β* variants

By analysing variants of the four genetic markers (*pvmsp-3α, pvmsp-3β, pvmsp*-1, and *pvcsp*) in combination, results showed high diversity of the parasites with 26 genotypic patterns in Hainan Province (Table 2). Almost all haplotypes were different in every isolate. The frequency of every haplotype was 3.7% with even distribution in almost all haplotypes except variant B with haplotype A of H3 and A1 of *pvmsp-3α*, PA2 of *pvmsp-3β*, Sal-1 of *pvmsp-1*, and VK210 of *pvcsp,* which had two members and had corresponding higher frequency(7.4%).

**Table 2:** **Combined haplotypes and frequencies of** ***pvmsp*** **-** ***3α*** **,** ***pvmsp*** **-3** ***β*** **,** ***pvmsp*** **-1, and** ***pvcsp*** **from 27 vivax isolates of Hainan Province**

**Allelic variants**	***pvmsp*** **-** ***3α***		***pvmsp*** **-3** ***β***	***pvmsp*** **-1**	***pvcsp***	**Numbers**
	***Alu*** **I**	***Hha I***	***Pst*** **I**			
A	A2	H1	PB1	R-H	G-VK210	3.70%
B	A2	H1	PU	S-F	E-VK210	3.70%
C	A2	H1	PU	S-A	E-VK210	3.70%
D	A6	H2	PA1	S-E	K-VK247	3.70%
E	A6	H2	PA1	S-C	A-VK210	3.70%
F	A6	H9	PU	B-I	A-VK210	3.70%
G	A6	H10	PA6	S-F	G-VK210	3.70%
H	A5	H11	PM	S-G	C-VK210	3.70%
I	A1	H3	PA2	S-A	F-VK210	7.41%
J	A1	H3	PA2	S-D	H-VK210	3.70%
K	B1	H7	PB2	S-D	J-VK247	3.70%
L	A1	H3	PA7	S-A	F-VK210	3.70%
M	A3	H2	PU	S-F	M-VK247	3.70%
N	A8	H6	PA5	R-H	G-VK210	3.70%
O	A4	H8	PA3	S-F	I-VK247	3.70%
P	A1	H3	PU	S-A	F-VK210	3.70%
Q	A1	H3	PA2	S-B	F-VK210	3.70%
R	A7	H5	PU	S-F	E-VK210	3.70%
S	A3	H4	PU	S-E	D-VK210	3.70%
T	A1	H3	PB3	S-A	F-VK210	3.70%
U	A3	H2	PA1	S-C	B-VK210	3.70%
V	A6	H2	PA1	S-C	A-VK210	3.70%
W	A1	H3	PA2	S-A	F-VK210	3.70%
X	A3	H2	PA1	S-C	A-VK210	3.70%
Y	A3	H2	PU	S-F	L-VK247	3.70%
Z	A3	H2	PA1	S-C	A-VK210	3.70%

A: The allelic type of *pvmsp*-*3α* was based on the fragment sizes of *AluI* -digested PCR products; H: The allelic type of *pvmsp*-*3α* was based on the fragment sizes of *HhaI-*digested PCR products; PA: The allelic type of *pvmsp*-3*β* was based on the A type fragment sizes of *PstI* -digested PCR products; PB: The allelic type of *pvmsp*-3*β* was based on the B type fragment sizes of *Pst I* -digested PCR products; PU: Undigest fragment; S: Sal-I type; B: Belem type; R: Recombination type of *pvmsp*-1.

## Discussion

Malaria is one of the most important infectious diseases in China, and most of the autochthonous malarial cases have been reported in the southern region of China in earlier decades, including Hainan Province and Yunnan Province. Parasites, including *P. falciparum* and *P. vivax*, are prevalent in those two provinces; however, in the past ten years, malaria has been controlled in China. The autochthonous malaria cases in the corresponding endemic regions have been reduced almost to zero with the support from the Chinese Government and the dedicated efforts of healthcare professionals. Hainan Province, one of the most endemic regions with historically high transmission of *P. falciparum*, has not reported any autochthonous *P. falciparum* malaria case since 2010 [16,17]. Moreover, no *P. vivax* autochthonous case has been reported since 2013 [18]. Information on genetic diversity of malarial parasites is important to understand the dynamics of disease transmission, to develop targeted anti-malarial drugs, and to develop effective methods to trace the origin of infections. Furthermore, knowledge of parasite population genetics would be useful in designing and monitoring strategies for elimination, and provide valuable metrics for monitoring the success of control efforts, if population genetic parameters accurately reflect transmission intensity. However, the information of autochthonous *P. vivax* population in Hainan Province is limited.

The *pvmsp*-1 locus codes for a major asexual blood-stage antigen had extensive polymorphism in isolates from different geographical regions. Three allele types including Belem, Sal-1, and recombinant variable block 5 have been found in isolates of *P. vivax* worldwide. The *pvmsp*-1 markers have been used in genetic studies of *P. vivax* in many countries, including India, Myanmar, Brazil, Latin America, and Korea [10,12,19,20]. In 2003, this marker was used for investigating 33 samples collected from various sites of *P. vivax* endemic areas in mainland China [21]. The result of this investigation showed that three allele types were found in China and that Sal-1 and recombinant allelic types were dominant, although the frequency of those types were different in different areas. In Hainan, three allele types were found and some sample had two allele types in 2002 and 2005 [21,22]. In the present study, three allele types were detected among the Hainan isolates, with the Sal-1 type being the predominant one. A similar degree of diversity was also found in Hainan Province in 2002 and 2005 [21,22]. Since 2003, some new subtypes have been found according to their amino acid sequences. In present study, the sequence of amino acid of *pvmsp*-1 was longer than most published sequences and most of subtypes were similar with other stains in phylogeny tree of *pvmsp*-1. Only subtype F and subtype I showed 100% identity with AAR30523 and AAR30526 strains isolated from Hainan in 2003, respectively. Comparison with published sequences, some of subtypes had single amino acid inserted or deleted or substituted and became new subtypes. Furthermore, Sal subtype I from Hainan isolates had some similarity with the subtype K and the subtype L, which were collected from Myanmar [12]. Those isolates were contained the amino acid sequence MKKELLDQYK specific to the Sal 1 type rather than the DKKLLKEYE specific to the Belem type. This kind of isolate was first reported in China.

Currently, *pvcsp* acts as an important gene marker and it has been used successfully in epidemiological studies of *P. vivax* malaria. This marker has two types: classic type (VK210) and variant type (VK247); the former, is known as classic type with obvious character of repeat motif as GDRAA/DGQPA within the amino acid tandem repeat region whereas, the latter is known as variant type with ANGAGNQPG amino acid repeats within the amino acid tandem repeat region. These two types have a worldwide distribution [23,24]. VK210 has been observed as predominant type in many countries, although VK247 was reported to be the predominant type previously [25-28]. In 2001, 384 samples were collected from 10 vivax-endemic provinces and the results showed that both types of *csp* genes were observed and that VK210 was the dominant type [29]. Since then, many research devote to investigate the ratio of two types in different regions of China but VK247 and mixed infections also have only been found in south China, including Yunnan Province and Hainan Province [30,31]. Moreover, in other endemic areas, including Anhui, Hubei, Guangxi, Guizhou, Guangdong, there was only the VK210 type. In present study, VK210 and VK247 still exist in Hainan but there were no mixed infections. The ratio between VK210 and VK247 decreased from 87:12 in control malaria stage in 2002 to 23:4 in elimination stage of present study [30]. There are no statistical differences between these data (χ^2^ = 0.747, *df* = 1, P > 0.05). The changes of ratio of two types of *csp* have influenced the decrease of malaria cases in Hainan Province. There is limited information concerning the sequence of the subtypes in the two types of *pvcsp* from China. Comparison with published sequences in NCBI and nearby sequences from phylogeny trees of *pvcsp*, most of subtypes were new alleles because they had different numbers of repeat motif or had different mutations in motif, except subtype C was showed 100% identity with AAV80840 strains isolated from Mauritania in 2004.

*pvmsp*-*3α*, *pvmsp*-3*β* and *pvmsp*-3γ are members of a multi-allelic diversifying selection to fit the evolution[31,32]. This gene family exhibits a high degree of genetic diversity but *pvmsp3* may be analogous with *pfmsp3*, not homologous [31]. The PCR-RFLP method has been applied for analysing the degree of diversity in *pvmsp*-*3α* and *pvmsp*-3*β* over many years [33,34], but this method has recently been suspected not to be suitable for broad geographic studies or tracking parasite populations [33]. The larger sample showed that some of identical haplotypes could be produced from analogous bands after PCR-RFLP analysis and revealed incongruence between the observed levels of nucleotide polymorphism and the pattern of PCR-RFLP haplotype [33].

Based on the length of PCR products, allelic type A and allelic type B of *pvmsp*-*3α* have been detected among the 27 tested isolates. The observation was different from a previous report from several locations in China, in which type A, B, C, and mixed infection were detected in *P. vivax* isolates from Sanya city of Hainan Province [7,35]. The lack of type C allele types in the present study could be due to a disappearance of this allele before implementation of the NMEP. Taking consideration of frequency of genotype, type A had a high frequency (96%, 26/27) and was a predominant type at elimination stage of malaria in Hainan Province. The present study results are consistent with previously published reports indicating type A as the most prevalent type around the world with a frequency 70 to 100% (average ~80%) in many regions of China and countries of Asia and South America [7,11,13,34].

In the analysis of *pvmsp*-*3α*, PCR-RFLP allele types and frequencies, 11 patterns were detected in the *pvmsp*-*3α* gene after digestion of the PCR products with *Hha* I, and the most frequent allele variant were H1 and H3 subtypes. The *Hha* I allelic types, including H1, H3, H4, H5 H6, H7, H8, H10, and H11 were found. Those *Hha* I allelic types had been found in other regions of China and in parts of the world. Similarly, variants H1, H3, H5, H7, and H10 were found in Myanmar [7]. The variants, including H4, H6, and H11 isolated in the present study, have high similarity with variants A3, A6 and A4 isolated in Thailand [36], respectively. Variant H8 has extensive similarity with variant A6 isolated in Brazil. In China, variants H1, H3, H6, H7, and H10 were reported earlier in Hainan Province [7] and allelic variants H4 and H11 were also identified in Anhui Province [35]. Others variants, including A9 and A11, were found only in Anhui Province. Moreover, variants A2, A3, A5, and A10 were not found in present study. However, new allelic variants H2 and H9 have been reported in the present study, which were not described in previous reports.

Nine patterns were detected in the Pv*msp*-*3α* gene after digestion of PCR products with *Alu* I, with PA1 pattern being the predominant in Hainan Province at the elimination stage of malaria. Some allele types of *Alu* I digestion are comparable to those reported in other parts of the world, such as allele types A2, A3, A4, and A5 isolated in Anhui Province of China [35], types A1, A2, A3, A4, A5, and A8 in Pakistan [11], types A1, A2, A3, A4, and A5 in Iran [37], and types A1, A2, and A5 in Colombia [38], which suggests that these allele types of *P. vivax* may have a global distribution. The allele variant B1 was a new allele identified in the present study. The analysis of Pv*msp*-*3α* gene marker suggested that *P. vivax* populations in Hainan Province showed diversity at elimination stage of malaria and shared the majority of allelic variants with other parts of China and the world.

*pvmsp*-3*β* gene encodes merozoite surface protein dominated by alanine-rich central domains, which is strongly predicted to form a coil-like tertiary structure. The structure of central domain is radically divergent with the majority bearing large insertion/deletion mutations and it has been employed as a molecular marker to evaluate genetic diversity of *P. vivax*. Only type A and type B alleles were detected among four types of alleles in *pvmsp*-3*β* marker at the elimination stage of malaria, with type A being more abundant (62.9%) than type B. In comparison with previous study on *P. vivax* isolates from Hainan Province in 2006 [7], the multiple genotypes of *pvmsp*-3*β* marker have been decreased from four to two types. In addition, in the present study, RFLP analysis revealed different allelic compositions and a total of nine alleles were identified. Interestingly, despite the differences in geographic regions, some allele types of *Pst* I digestion were comparable to those reported in other parts of the world, such as allele types PA1, PA3, PB1, and PB2 isolated in China and Myanmar [7], type PA5 in Pakistan [15], types PA2, PA3, PB1, and PB2 in China and Thailand [35], suggesting that these allele types of *P. vivax* may have a global distribution. In the present study, new allele variants PA1, PA4, PA5, PB3, and PM were identified. The levels of mixed-genotype infections were correlated with the levels of endemicity and the power of sensitivity on selected gene marker. The discrimination of *pvmsp*-3*β* gene marker for mixed-genotype infections in several endemic regions has more sensitivity than other markers, especially in endemicity; for example, genotyping the Thai samples for *pvmsp*-3*β* detected 20.5%, whereas combination with genotyping *pvmsp*-*3α* increased the mixed infection level to 29.5% [33]. On the other hand, genetic diversity of the malarial parasites is associated with the levels of endemicity and transmission intensity [15]. In hyperendemic areas, such Myanmar [12], and Thailand [39], *P. vivax* is highly diverse with multiplicity of infections. However, in Korea and Iran [23,40], *P. vivax* was resurgent, and mixed infections were detected; however, the infection proportion was very low. In the present study, mixed infection was still detected by *pvmsp*-3*β* at the elimination stage of malaria. This information could suggest that the genetic diversity of the malaria parasites is not only associated with the levels of endemicity and transmission intensity, but could also be associated with epidemic times in history.

*pvmsp*-3α and *pvmsp*-3*β* are promising markers for epidemiological applications. Compared with the simple PCR-RFLP method, sequencing of these markers may offer significantly higher power for determining parasite genetic diversity. Analysis of diversity in *pvmsp*-3α and *pvmsp*-3*β* by sequencing opens new perspectives for diversity analysis and this method had been used at the Thai-Myanmar border area and revealed that the extent of allelic diversity in *P. vivax* populations in Thailand [41].

## Conclusion

The present study indicates that there was still a high degree of genetic diversity of P. vivax in Hainan Province at the pre-elimination stage, with 26 unique haplotypes observed among 27 samples, inferring that *P. vivax* populations are more resilient to elimination, compared to Solomon Islands and Thai-Myanmar border areas.
